# Social Representativeness and Intervention Adherence—A Systematic Review of Clinical Physical Activity Trials in Breast Cancer Patients

**DOI:** 10.3389/ijph.2024.1607002

**Published:** 2024-05-09

**Authors:** Ragna Stalsberg, Monica Dahle Darvik

**Affiliations:** ^1^ Department of Circulation and Medical Imaging, Norwegian University of Technology and Science (NTNU), Trondheim, Norway; ^2^ Department of Neuromedicine and Movement Science, Norwegian University of Technology and Science (NTNU), Trondheim, Norway

**Keywords:** sampling bias, social validity, research equity, exercise trials, breast cancer, mammae cancer, meta-analysis

## Abstract

**Objectives:**

Representativeness in physical activity randomised controlled trials (RCT) in breast cancer patients is essential to analyses of feasibility and validity considering privileged- social groups. A step-by-step exclusion of less privileged groups through the trial process could reinforce health inequality. This study aimed at examining representativeness in breast cancer (BC) physical activity trials, investigate associations between socio-economic status (SES) and intervention adherence, and explore associations between representativeness and the relationship between SES and intervention adherence.

**Methods:**

Systematic, computerised searches were performed in PubMed, CINAHL, AMED, EMBASE and PsycINFO. Additional citation-based searches retrieved 37 articles. Distributions of education level, ethnicity, and marital status in study samples were compared to national populations data to estimate representativeness in less privileged groups.

**Results:**

A preponderance of studies favoured educated, married and white patients. Only six studies reported SES-adherence associations, hampering conclusions on this relationship and possible associations between representativeness and an SES-adherence relationship.

**Conclusion:**

Less educated, unmarried and non-white individuals may be underrepresented in BC physical activity RCTs, while SES-adherence associations in such trials are inconclusive. Unintentional social misrepresentations may indicate that disguised inequity warrants revived attention.

## Introduction

Clinical trials in breast cancer (BC) research have contributed significantly to improved treatments, health-related quality of life, and survival probability for BC patients [1]. For example, numerous clinical physical activity (PA) trials have been conducted. Nevertheless, faced with the reality of social health inequalities [2, 3], it is essential to ensure that the results of these trials benefit all, not exclusively the most privileged. Previous analyses have concluded that study samples only slightly represent real-world patient populations [4] due to participants having better health status, lower age, and a dominant ethnicity. However, research inequity on the part of socioeconomic status (SES)-groups has been scarcely investigated. As PA appears to improve physical fitness [5–8], fatigue [5, 6, 9, 10], and physical functioning [5, 9, 11] in BC patients and survivors, knowledge about representativeness across SES and privilege in randomised controlled trials (RCT) with PA is warranted.

Privilege refers to (often unrecognised) advantages that benefit people belonging to certain groups based on factors like their SES, ethnicity, age, gender identity [12]. High-SES individuals, for example, are considered to have health benefits as a result of their belonging to a privileged social group [13]. SES, is ideally measured as the combination of an individual’s or groups’ education, income and occupation [14] and it is often used to examine social health inequalities [15]. In the case of BC, there is an excess risk associated with increased education, in both men [16] and women [17]. Unmarried women, who face a multitude of hardships and less privilege [18], may have a higher risk of developing BC [19]. Similarly, less privileged non-whites have higher odds of being diagnosed with aggressive BC [20]. However, BC in men and less privileged women is generally diagnosed later, making these cases more severe compared to BC diagnosed in privileged women [21]. Despite higher incidence rates of early BC, higher survival rates and better health-related quality of life after diagnosis [22] are associated with higher education [23]. In young men with invasive BC, overall survival is found to decrease in groups living in ZIP-code areas associated with low SES [24]. Presumably because <1% of all BC incidents occur in men [25], most PA research concern women. Female patients often report weight gain, which is associated with undesirable BC-outcomes [26–28]. Thus, it is often recommended that they pursue regular PA [29]. An inactive lifestyle is suggested to be a significant social determinant of decreased BC survival probability in low-SES groups [30], and according to Boer et al., there is strong evidence that BC recurrence and mortality are strongly associated with leisure-time PA (LTPA) due to the biological mechanisms affected [31].

PA is defined as any bodily movement produced by skeletal muscles that results in energy expenditure [32] and includes the active transport-, leisure time- (including exercise), job-related-, and household PA domains [33]. The relationship between PA and the risk of BC is well documented [34–44] and is evident for LTPA in postmenopausal BC [45, 46]. However, it has been found that different SES-groups in general are inclined to different domains of PA, with high-SES groups being more physically active within the LTPA domain [47]. Comparable results are reported in studies of women with BC [48–51]. Hence, for RCTs with LTPA, there is a risk of selection bias, which is also acknowledged in the Exercise guidelines for cancer survivors: “*…the individuals enrolled in studies commonly meet prespecified eligibility criteria (…) and were willing to take part in research. This often results in a sample that is healthier or with higher physical function and exercise motivation that may not fully generalize to the broader population of cancer survivors.*” [52].

Moreover, if there are SES-differences in PA among women with BC in general, participation and adherence to PA trials may differ accordingly. Studies have shown different PA barriers across SES [53, 54], partly explaining variances in activity levels [55–59]. If low-SES individuals perform less LTPA than high-SES individuals [47], the former may be prone to lower participation and adherence rates in exercise RCTs. In addition to findings of better exercise trial adherence in high-SES groups [60, 61], less privileged individuals less frequently volunteer to participate in research [62, 63], due to barriers to access [62], lower health literacy and negative attitudes towards research, additional costs, or disease status [64]. These findings may imply that RCTs with which a privileged group is more familiar and motivated for the provided intervention (i.e., LTPA/exercise) run higher risks of selection bias than RCTs for which compliance is less affected by social grouping.

Hence, the external validity of LTPA trials for BC patients may be impaired, albeit unintendedly, by a stepwise exclusion of less privileged patients; they appear to be recruited and participate less frequently, and may also have lower adherence rates [65]. The general view is that RCT samples must be homogeneous to gain internal validity and reliable results [66, 67]. Simultaneously, the declarations of Helsinki [68], legitimately and necessarily, prevents researchers from obliging participation for the purpose of representativeness. Consequently, social biases may be fortified [64, 65].

The credibility and success of medical progress depend on transparent reporting [69]. Thus, the CONSORT guidelines developed to improve RCT-articles [70]. Nevertheless, participant attributes, such as SES-indicators, are seldomly reported [71, 72], impeding successful assessments of equitable research [72]. A reasonable alternative may be to employ the available variables that indicate, or strongly correlate with, privilege and SES. Previous reviews of PA-SES associations have found that education is the most reported SES-indicator [47, 73, 74]. Furthermore, frequently reported characteristics, such as ethnicity and marital status, both reflect social privilege and correlate with SES, thus they may be considered fair representations of privilege in the absence of precise SES-indicators.

The objective of this systematic review was to study previous PA RCTs on BC patients with the intention of examining a) SES-related information and representativeness, b) associations between SES-related indicators and PA intervention adherence, and c) associations between representativeness and reported relationships between SES and intervention adherences. There are, of course, real obstacles to recruting less privileged patients, so non-representative samples should not be seen as researchers’ unwillingness or lack of effort to include the less privileged. The overall aim of the study was not to evaluate single studies conducted for purposes other than representativeness *per se*, but rather to highlight disguised patterns across comparable studies [75]. Furthermore, the aim was not to provide meta-analyses and precise sample- vs real-world ratios; however, the study may render a departure point for improving representativeness on the part of less privileged groups.

## Methods

**FIGURE 1:**
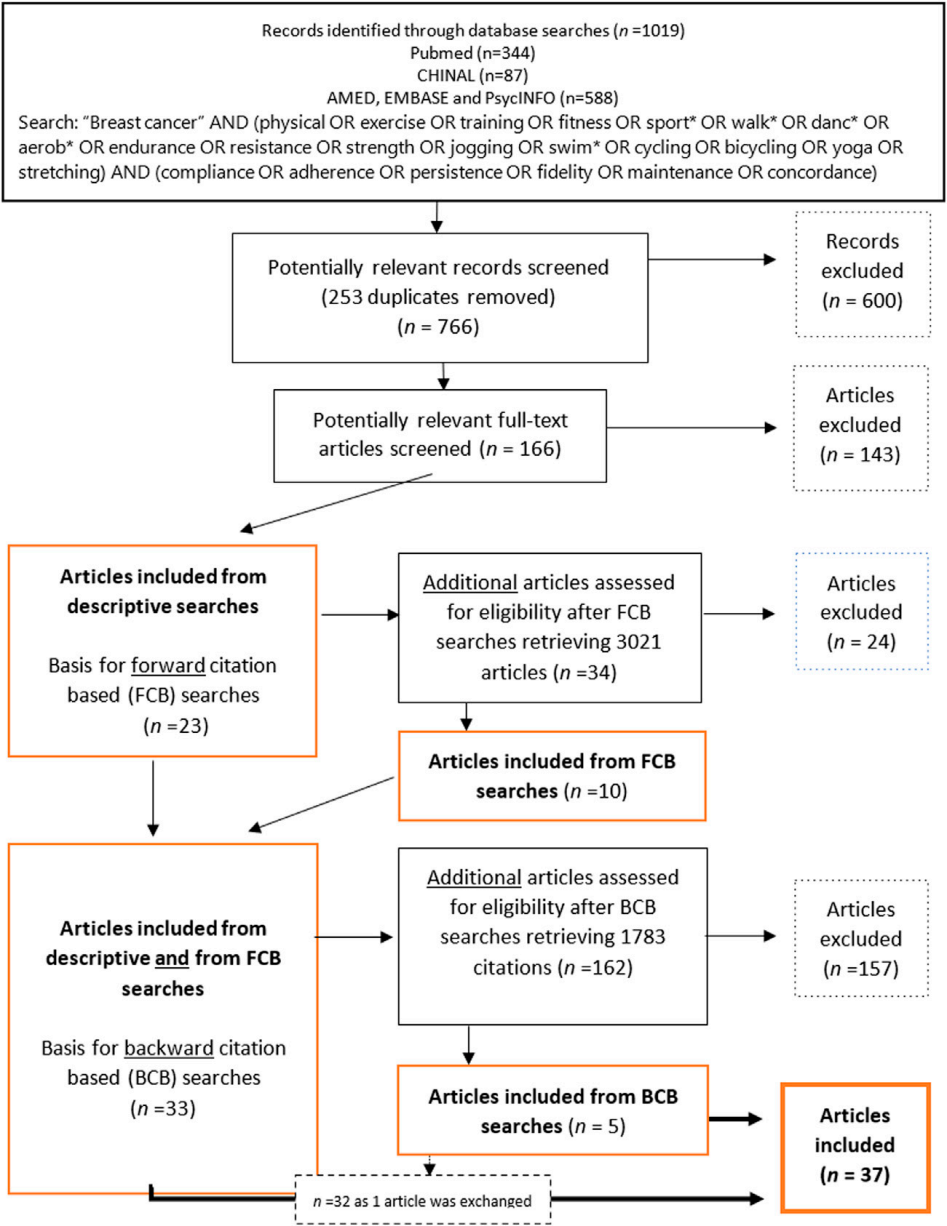
Flow chart of inclusion and exclusion of articles through descriptive- and citation-based searches (Global, 2000–2020).

Systematic, computerised searches were conducted in the PubMed, CINAHL, AMED, EMBASE and PsycINFO databases. The first round followed a traditional method [76]; we specified a search query by a set and combinations of words, and all publications indexed in the databases that contained those words were returned. “Compliance,” “persistence,” “fidelity,” “maintenance” and “concordance” were used as synonyms for the variable “adherence.” Similarly, “physical,” “exercise,” “fitness,” “sport” and “training” were used as synonyms, in addition to exercise-specific terms, such as “dance,” “swim,” “walk” and “yoga,” to cover any type of PA intervention. An asterisk (*) was attached to the roots of the words to broaden the search, and to retrieve variations on these terms. Because the terms are used interchangeably in the literature, both “*patients*” and “*survivors*” were used to cover relevant study samples. As recommended by Bramer et al. [77]. Boolean combinations were used to construct suitable search queries in combination with the basic term “breast cancer” (see Figure 1). There were no search limits as to publication year.

The following criteria guided the inclusion and exclusion process, defining the final dataset:

### Inclusion Criteria

a) Empirical studies
b) …reporting RCTs including a PA intervention.
c) …including individuals undergoing BC treatment within one-year postdiagnosis.
d) …reporting adherence to the PA intervention.
e) …presenting a comprehensible calculation of adherence.
f) …written in English.

### Exclusion Criteria

a) Reviews, theoretical and descriptive papers, books, theses, letters to editor or editorials.

Articles:

b) …reporting on multiple cancer diagnoses (even if BC was included).
c) …not reporting any SES-related characteristics of the sample … written in any non-English language.

The descriptive searches retrieved 1,019 articles. After excluding immediate duplicates, 766 articles were manually examined and deemed potentially relevant based on a screening of titles and abstracts. This left, 166 potentially relevant articles that were examined to establish their eligibility according to the criteria. An algorithm for all variables was implemented using Python3 to ensure the eligibility of the articles selected. In total, 143 articles were excluded for a total of 23 remaining articles (Figure 1).

To retrieve all possible relevant studies, thus reducing the risk of missing information [78], citation-based searches (described by Hu et al. [79], but implemented in Rysstad and Pedersen [80] and Darvik et al. [81]) were performed based on the 23 articles found in the descriptive searches. A forward citation-based (FCB) search of articles that cited the articles included in the descriptive searches was first reviewed for eligibility, and 34 potentially relevant articles were identified, 24 of which were excluded. A backwards citation-based (BCB) search, where the reference lists in the 33 articles already included in the material were scanned for eligible studies, retrieved 5 articles of which one replaced a previously included article, following the inclusion criteria.

In cases in which an article reported on two different parent trials, the article was included on the condition that at least one of the parent studies met inclusion criteria c. If two articles were reporting on the same parent RCT sample and overlapped in their data, the earliest article was included. If, however, only one of them reported adherence rates, the article omitting adherence rates was excluded. Nevertheless, the included article had to report on SES-related patient characteristics. In the subsequent step of the inclusion/exclusion process, only articles reporting the samples’ SES distributions were included for further analyses.

The second author performed the database searches but conferred with the first author to discuss any cases of doubt about the potentially relevant articles returned from the query.

### Data Extraction and Analysis

The following variables were extracted from the articles: sample descriptions, intervention designs, aims of the studies, measures of SES or privilege, their distributions, and adherence calculations and -rates. For studies that investigated the associations between adherence and SES-related factors, the reported results were registered.

The SES-related distribution in the sample was compared with the corresponding distribution within the country in which the study was conducted, at the matching time of publication. Because adherence to intervention protocols was embedded in our study objectives, only intervention group characteristics were analysed. Due to third-party researchers’ limited access to patient population data, SES-related distribution in a country population, in the corresponding age groups, was used as a proxy for the patient population. For the education variable, the percentage of the study sample holding any tertiary education (no degree required) was compared with the percentage of the country population holding the same educational level (i.e., all formal postsecondary education, including public and private universities, colleges, technical training institutes, and vocational schools [82]), using public statistics for reference [83–86] (see Supplementary Files S2, S3 for details). Because the final data set included articles about women exclusively, all statistics retrieved and analysed, were relevant to women.

Similarly, the proportion of married women in the study samples and in the associated country populations was compared using United Nations World Marriage Data (age 45–49) [87] and a corresponding analysis for ethnicity based on national censuses [88–92]. Frequencies in the *white alone* and *non-indigenous and non-visible minority* categories from accessible censuses were used to estimate figures for intercensal years by interpolation. The relative differences between the study samples and the associated countries were calculated as follows:

$$\frac{\%\;with\;high\;SES\;in\;study\;sample-\%\;with\;high\;SES\;in\;country\;population}{\%\;with\;high\;SES\;in\;study\;sample}*100$$

Studies reporting on the association between SES-related factors and adherence to the PA intervention were further explored, and variations in adherence calculations were examined.

## Results

The preliminary screening excluded 20%–25% of all potentially relevant articles due to a lack of information about SES-related characteristics. The subsequent selection process, following descriptive, FBC- and BCB-searches, retrieved 37 eligible articles (Figure 1).

A total of 28 (76%) of the included studies were published between 2010 and 2020, and the oldest article was published in 2002 [93]. Most studies (81%) were conducted in Western countries: 18 in the United States, 5 in Canada, two in the Netherlands, and one each in Spain, France, the United Kingdom, Denmark and Sweden. Six (16%) studies were Asian, including one Indian, two Chinese and three Taiwanese studies. In addition, one Brazilian study was included. The (full) sample sizes ranged from 14 [94] to 301 [95], including 11 studies with <50 participants, 15 studies with 50–96 participants, and 11 studies with 100–301 participants. The mean age ranged from 42.1 to 63.2 years (total mean = 51 years) (Table 1). All included studies were of female BC patients.

**TABLE 1:** Study aim, sample, SES and intervention descriptions, adherence formula, and adherence outcome in included studies. (Global, 2000–2020).

Study	Aim (short form)	Intervention group	Measures of SES	Intervention (PA part) description	Adherence calculation	Adherence outcome (PA-part)
Al-Majid et al., 2015 [94]	To examine an exc. intervention, the effects of exc. on HB and vo2 max and their association with changes in CRF and QoL, and changes in infl. markers	N = 7 Mean age: 47.9 BC stage: I-II United States	Education + Income - Ethnicity + Marital status + Employment status +	9–12-week: Supervised treadmill exc. w/progressive increase in workload, 2–3 times/wk for the duration of chemotherapy	# exc. sessions completed as per protocol out of the total # of scheduled sessions	95%–97%. All P completed remaining sessions per protocol
Anderson et al., 2012 [96]	To determine the effect of a moderate, tailored exc. program on health-related quality of life, physical function, and arm volume	N = 52 Mean age: 53.6 BC stage: I-III United States	Education + Income + Ethnicity + Marital status - Employment status -	2 exc. sessions/wk including 30 min mod/hard walking, 20 min upper and lower body strength training, and 10 min of stretching	Weekly visit attendance	P completed 71.2% of all sessions (range 0%–97%). 61% attended >75% of sessions and 13% attended <50%
Arem et al., 2016 [97]	To describe the intervention, report 6- and 12-month exc. adherence and cardiorespiratory fitness changes and identify predictors of exc. adherence	N = 61 Mean age: 62 BC stage: I-III United States	Education + Income - Ethnicity + Marital status + Employment status -	1 year: combination of a twice/wk supervised aerobic and resistance training program. Brisk walking ≥150 min/wk. 6 exc. performed for 8–12 repetitions for 3 sets	Self-recorded type, duration, and average HR. Attendance to sessions. Goal of 150 min/wk of aerobic exercise	An average of 115 and 119 min/wk aerobic exc. at 6 months. ≈50% reported ≥120 min/wk, >30% reached 150 min/wk. An average of 72% and 70% of strength sessions
Ariza-Garcia et al, 2019 [98]	To evaluate the effectiveness of a web-based exc. program	N = 34 Mean age: 48.8 BC stage I-IIIA SPAIN	Education + Income - Ethnicity - Marital status + Employment status +	8-week 3 sessions/wk. Aerobic exc. intensity between 45% and 60% of max HR, 15–30 min. A total of 5 strength exc. of low intensity with functional implementation	A ratio of # of sessions performed out of # of sessions prescribed	Adherence rate for the e-Cuidate Chemo group was 73.33%
Bland et al., (2019) [99]	To investigate the effect of a combined supervised and home-based exc. intervention	N = 12 Mean age: 49.5 BC stage I-III CANADA	Education + Income - Ethnicity + Marital status + Employment status -	8–12-week: Immediate exc. (IE) during chemotherapy or delayed exc. (DE) after chemotherapy. Supervised aerobic, resistance, and balance training: 3 days/wk	Any deviation from plan was considered as adherence not met.	Adh. to supervised aerobic exc: 77% ± 30% and 78% ± 24% for, IE, and 81% ± 17% and 81% ± 21% for DE. Adh. to resistance and balance exc: 78% ± 37% for, IE and 93% ± 6% for DE.
Cadmus et al, 2009 [100]	To determine the effect of exc. on quality of life	N = 25 Mean age: 54.5 BC stage: 0-IIIA United States	Education + Income - Ethnicity + Marital status - Employment status -	6 M: Home-based exc. program	Average min/wk of mod-intensity aerobic exc. performed from 0 to 6 months. (Good: 80% of prescription)	P performed 144 (SD = 75) min of activity/wk (range: 0–253). 64% met the goal of 150 min/wk. P returned 23.1 wkly logs, with 72% returning all logs
Carayol et al., 2019 [101]	To assess the 1-year follow-up effects of an exc.-diet intervention	N = 72 Mean age: 51.2 BC stage: I-IIIC FRANCE	Education + Income - Ethnicity - Marital status + Employment status +	26-week: 3 exc. sessions/wk, combining one muscle strength session and two aerobic sessions/wk	Log with whether sessions were achieved or not, # achieved exc., duration, and rating of perceived exertion	Adherence was 67% of completed planned sessions in the exc. components
Chandwani et al., 2010 [102]	To examine the effects of yoga on quality of life and psychosocial outcomes	N = 30 Mean age: 51.39 BC stage 0-III United States	Education + Income - Ethnicity + Marital status + Employment status +	6-week of RT: Up to two 60-min yoga classes/wk	# Of participants who declined and class attendance	50% attended all 12; 28% attended 11; and 3% attended 10 classes. 50% had perfect attendance and 81% attended more than 80% of the classes
Chen et al., 2013 [103]	To examine the efficacy of a qigong intervention on quality of life	N = 49 Mean age: 45.3 BC stage: 0-III CHINA	Education + Income + Ethnicity - Marital status + Employment status -	5 or 6-week of RT: Five 40-min qigong classes/wk. Encouraged to practice qigong when not with qigong master, and after RT.	Attendance rates %	30.4% P attended 100% of sessions, 65.2% attended ≥80% of sessions, 78.3% attended 50% of sessions, and only 13% attended<20%
Courneya et al., 2013 [95]	To compare two different doses and types of exc. for improving physical functioning and symptom manage	N = 101 + 104 Mean age 50.3 BC stage: I-IIIC CANADA	Education + Income + Ethnicity + Marital status + Employment status +	3 times/wk during chemotherapy: either 25–30 min aerobic exc., 50–60 min aerobic exc., or 50–60 min aerobic and resistance exc	Frequency and volume (min) of aerobic exc	Standard, High, and Combined group completed 87.8%, 81.6%, and 78.0%, respectively
Courneya et al., 2008 [104]	To identify the key predictors of supervised exc. training during the trial	N = 160 Mean age: 49 BC stage: I-IIIA CANADA	Education + Income + Ethnicity - Marital status + Employment status +	12–24-week. AET: 3 times/wk cycle ergometer, treadmill, or elliptical trainer. Progressing. Resistance: 3 times/wk 2 sets of 8–12 repetitions of 9 exc. at 60%–70% of their estimated 1-repetition max	If >95% of the sessions were done at the appropriate duration and intensity: % attendance, based on length of chemotherapy (12–24 Wk)	Adherence was 72.0% T 30.1% and 68.2% T 28.4% in the AET and RET groups, respectively (*p* = 0.411), with 51.2% achieving 80% attendance (range, 0%–100%)
Demark-Wahnefried et al., 2008 [105]	To assess feasibility and the impact of two home-based interventions	N = 29 + 32 Mean age: 42.1 BC stage: I-IIIA United States	Education + Income - Ethnicity + Marital status + Employment status -	Aerobic exc. ≥ 30 min/day ≥3 times/wk and to perform strength training exc. every other day. Instructions provided. 2 sets of stretches and 7 strengths exc	Completion rates	CA + EX sessions/wk, mean (SD): 3.2 (1.1). Aerobic min/session, mean (SD); % who exc. ≥3times/wk ≥ 30 min/session: 43%. Strength training, mean (SD); % who completed 7 exc.: 8%
DeNysschen et al., 2011 [123]	To examine the nutritional symptoms and body composition outcomes of aerobic exc	N = 36 + 30 Mean age: 49 BC stage: I-III United States	Education - Income + Ethnicity + Marital status + Employment status +	Cardiovascular exc., 3–5 times/wk, T1-T2: 20–30 min, T2-T3: 30 min/session at min. and Borg scale >12–14, mod exertion	a) To protocol: frequency, duration and intensity questions. b) Changes in cardiorespiratory fitness: VO2peak and METs	P adherence was 74% for EE group at end of chemotherapy and 78% at the end of the study, CE group adherence was 86% at the end of the study
Dieli-Conwright et al., 2018 [124]	To assess whether exc. can attenuate adipose tissue inflammation	N = 10 Mean age: 53 BC stage: I-III United States	Education - Income - Ethnicity + Marital status - Employment status -	16-week supervised exc. sessions 3 times/wk. Days 1 and 3 were resistance and aerobic exc. approx. 80 min, Day 2 aerobic exc. approx. 50-min	Rates of session completion	P completed on average 46 of the 48 exc. intervention sessions
Gokal et al., 2016 [106]	To assess the effectiveness of a self-managed physical intervention in improving psychosocial health outcomes and levels of PA	N = 25 Mean age: 52.08 BC stage I-III UNITED KINGDOM	Education + Income - Ethnicity - Marital status + Employment status +	12-week home-based, self-managed, mod. intensity walking	Completion of intervention. Patients were provided with a diary to keep a log of walking duration and intensity	80% of P adhered to the intervention and completed walking diaries
Huang et al., 2015 [107]	To identify the trajectory of exc. adherence and its predictors	N = 78 Mean age: 48.27 BC stage: I-IIA TAIWAN	Education + Income - Ethnicity - Marital status + Employment status +	12-week. Walking program. 3 times/wk Wk 1–6, 5 times/wk Wk 7–12. 15–25 min/session Wk 1–4, 25–35 min/session Wk 5–8, 35–40 min/session Wk 9–12	Time (duration/session and frequency/wk) and intensity (the ratio of the highest HR during exc. to the target HR)	The mean time adherence: 87.1%. Highest in wk 3 (99.4%), lowest in wk 11 (74.0%). The overall mean intensity-adherence rate was 97.59% (range = 95.14–99.18)
Kim et al., 2006 [125]	To examine cardiopulmonary responses to a moderate-intensity aerobic exc., assess adherence to exc.; and examine levels of overall PA 16 Wk postintervention	N = 22 Mean age: 51.3 BC stage: 0-III United States	Education - Income - Ethnicity + Marital status + Employment status -	8–24-week. Mod intensity supervised aerobic exc. program 3 times/wk. After completion, encouraged to continue their exc. at home or in a community setting	Frequency, duration, and duration at target HR. Exc.: # exc. sessions completed at prescribed level divided by # sessions prescribed	Overall adherence to exc. intervention was 78.3%–20.1%, but wk-to-wk variations ranging from 68.3% at wk 7%–95.0% at wk 3
Kirkham et al, 2018 [108]	To assess reach, effect-iveness, maintenance, and implementation of an evidence-based exc. and healthy eating program	N = 73 Mean age: 50.8 BC stage: I-III CANADA	Education + Income - Ethnicity + Marital status + Employment status +	20-week: Goal of 150 min/wk of mod-intensity aerobic exc., and resistance training. Supervised, 3 times/wk during adjuvant therapy + a step-down in supervised sessions	# exc. (and healthy eating) sessions attended/# prescribed sessions for all participants who started the program	Average attendance during treatment, posttreatment, and maintenance phases were 60% ± 26%, 52% ± 33%, and 50% ± 38%, respectively
Lee et al., 2019 [126]	To determine whether an 8-week high intensity interval training intervention prescribed using peak power output is feasible	N = 15 Mean age: 49.1 BC stage: I-III United States	Education - Income - Ethnicity + Marital status - Employment status -	8-week supervised 3 times/wk. 7 times of a 1-min interval at 90% peak power followed by a 2-min interval at 10%. 5-min warm-up at 10% followed by 20-min of protocol	Average min/wk of exc. over 8 Wk and % of sessions	Sessions attended was 82.3%. Overall, 80% P met both criteria; attended 19.2 ± 2.1 of 24 sessions and completed an average of 78 ± 5.1 of 90 min/wk of exc. over 8 Wk
Lund et al, 2019 [109]	To examine adherence to an intervention combining supervised and home-based exc	N = 62 (74) Mean age: ≈60 BC stage unreported DENMARK	Education + Income - Ethnicity - Marital status + Employment status +	50-week: Resistance exc. 20-week combined supervised/home based exc. followed by 30-week self-administered exc	Numbers of exc. sessions performed divided by expected # exc. sessions with >2/3 categorized as high adherence	# Of P w/high adherence to supervised exc. in late period compared to the early period: 65% vs. 48%. The % of participants with high adherence to home-based exc. was ≈55%
Matthews et al., 2007 [110]	To evaluate the effectiveness of a home-based walking intervention and quantify changes in PA, as well as in body weight and composition	N = 22 Mean age: 51.3 BC stage: I-III United States	Education + Income - Ethnicity + Marital status - Employment status +	12-Wk. Home-based, walking. Counselling visit (30 min) followed by up to 5 phone-counselling calls in wk. 1, 2, 4, 7, and 10 after randomization (10–15 min/call)	The % of sessions completed relative to # sessions recommended	Thirty-four of 36 women randomized (94%) completed the study. Overall adherence was 94%
Mijwel et al., 2018 [111]	To compare the effects of two training interventions on CRF, HRQoL, and symptoms	N = 74 + 72 Mean age: 53.5 BC stage: I-IIIA SWEDEN	Education + Income - Ethnicity - Marital status + Employment status +	16-week: 60 min x 2 sessions/wk in an exc. clinic. 5-min warm up; both resistance and high-intensity interval aerobic exc.; 10-min cool down	# Patients who completed 90% of sessions according to plan, divided by total # patients in the intervention groups	Adherence to the training program was 83% in the RT–HIIT group and 75% in AT–HIIT group
Mock et al., 2005 [112]	To determine the effects of exc. on fatigue levels during treatment	N = 60 Mean age: 51.3 BC stage: 0-III United States	Education + Income - Ethnicity + Marital status + Employment status -	A home-based mod-intensity walk exc. program for the duration of the treatment. Mod. walk 5–6 times/wk (≈50–70% of max HR). Brisk 15-min walk, increased to 30 min	85% of min. prescription: engaging in ≥60 min of aerobic activity weekly for ≥2/3 of the duration of the trial	72% adhered to the exc. prescription. Slightly better in those receiving chemotherapy (75%) than in those receiving RT (71%)
Paulo et al., 2019 [113]	To evaluate the impact of an exc. program on quality of life	N = 18 Mean age: 63.2 BC stage: I-III BRAZIL	Education + Income - Ethnicity - Marital status + Employment status +	9-M. Supervised sessions 3 times/wk. A combined training program w/resistance training (40 min) followed by aerobic training (30 min)	% Attended classes	Adherence to the combined training was 83%
Pickett et al., 2002 [93]	To describe adherence to a brisk walking intervention, examine the effects of symptoms and side effects on activity levels…	N = 23 Mean age: 48 BC stage: I-IIIA United States	Education + Income - Ethnicity + Marital status + Employment status +	Brisk walk for 10–15 min/day ≥5 days/wk at 60%–80% of max HR. Advanced to 30-min walking sessions 5–6 days/wk when possible. Throughout the course of therapy	The level of participation achieved in a behavioral regimen once the individual has agreed to undertake it	33% of the exc. group did not exc. at the prescribed levels
Ratcliff et al., 2016 [114]	To examine moderators and mediators of a previously reported 3-arm yoga RCT	N = 53 + 56 Mean age: 51.7 BC stage: 0-III United States	Education + Income + Ethnicity + Marital status + Employment status +	6-Wk Yoga program: warm-up/breathing; selected postures; deep relaxation; alternate nostril breathing/pranayama; meditation	% Attended classes	87% of YG and 85% of ST participants attended ≥12/18 classes
Reis et al., 2013 [115]	To compare a 12-week exc. Nia program practiced at home to usual care on fatigue, QoL, aerobic capacity, and shoulder flexibility	N = 22 Mean age: 54 BC stage: I-III United States	Education + Income - Ethnicity + Marital status + Employment status +	12-Wk Nia (cardiovascular and whole-body conditioning program of martial arts, dancing, and healing arts): 20–60 min 3 times/wk	Adherence to group assignment and activity instruction was assessed by reviewing participant logs	12 out of 22 P practiced at least 13 times for 12 Wk. At 12 Wk, P reported performing NIA 0–34 times, an average of twice/wk
Rogers et al, 2009 [116]	To determine the feasibility and effectiveness of a PA behavior change intervention to address social cognitive theory constructs	N = 21 Mean age: 52 BC stage: I-IIIA United States	Education + Income + Ethnicity + Marital status - Employment status -	12-Wk program. Goal: 150 min of moderate walking per wk. 12 individual supervised exc., and 3 individual home-based	% Completed exercise sessions	Participants completed 100% of the individual sessions, 95% of the individual update sessions, and 98% of the group sessions for an overall 99% adherence
Stan et al. 2012 [127]	To examine the feasibility of a Pilates program and the impact on physical and psychological parameters	N = 15 Mean age: 49 BC stage: I-IIIA United States	Education - Income - Ethnicity + Marital status - Employment status -	12 Wk of Pilates exc., 45-min Pilates exc. twice/wk for the first 4 Wk, 3 times/wk for the next 4 Wk, and 4 times/wk for the last 4 Wk	Accrual, retention, attrition, and treatment acceptability	74% of the recommended sessions were attended. 54% performed 75% or more of recommended sessions
Swenson et al., 2009 [128]	To compare the effects of intravenous zoledronic acid versus prescribed PA on changes in bone mineral density	N = 36 Mean age: 46.9 BC stage: I-III United States	Education - Income - Ethnicity + Marital status - Employment status +	12-M: Home-based walking program. P advised to reach a goal of at least 10,000 steps/day	Exc. log to record daily steps, adherence to the prescription of 10,000 steps each day	Adherence to the prescription of 10,000 steps/day was 75%, 86% at 3 months, 93% at 6 months, 96% at 9 months, and 93% at 12 months
Travier et al., 2015 [117]	To examine the effects of an exc. intervention on preventing an increase in fatigue	N = 102 Mean age: 49.7 BC stage: M0 NETHERLANDS	Education + Income - Ethnicity - Marital status + Employment status -	18-Wk: 2 × 60 min supervised aerobic and strength sessions/wk. Aerobic and muscle strength training (25 min each)	% Participation of classes offered	Patients in the intervention group participated in 83% of the classes offered
Vadiraja et al., 2009 [118]	To compare the effects of an integrated yoga program with brief supportive therapy	N = 42 Mean age: 46.7 BC stage: II-III INDIA	Education + Income - Ethnicity - Marital status + Employment status +	6-week. Yoga sessions, 60 min/day A combination of a set of breathing exc., meditation and yogic relaxation	Attendance rates	29.7% attended 10–20 sessions, 56.7% attended 20–25 sessions and 13.7% attended >25 sessions
Vallance et al., 2016 [119]	To examine the effects of a broad-reach PA behavior change intervention	N = 49 Mean age: 52.8 BC stage: I-IIIA CANADA	Education + Income - Ethnicity + Marital status + Employment status +	4–6 M: PA resource kit w/PA print materials, a step pedometer, and a step logbook. P wore pedometer daily during treatments	How many days the participant wore their pedometer during the primary study period	P wore pedometer for 85.2 days (range, 35–144 days; SD = 26.4) over a possible 90.1 days for an overall 95% adherence rate
van Waart et al., 2015 [120]	To evaluate the effectiveness of a home-based PA program and a supervised resistance and aerobic exc. program on fitness and fatigue	N = 76 + 77 Mean age: 50.2 BC stage: I-III NETHERLANDS	Education + Income - Ethnicity - Marital status + Employment status +	From 1. cycle of Chemo - 3 week after last cycle: 2 sessions/wk. 6 large muscle groups, 20 min/session. 30 min aerobic exc. Encouraged 5 days/wk, 30 min/session	# Sessions attended	On average, participants in OnTrack attended 71% of the planned sessions
Wang et al., 2011 [121]	To test the effects of a walking program on Taiwanese women newly diagnosed with early-stage BC.	N = 35 Mean age: 48.4 BC stage: I-II TAIWAN	Education + Income - Ethnicity - Marital status + Employment status +	6-week: walking program. Low- mod. intensity, 3–5 sessions/wk ≥ 30 min/session or 3 × 10-min sessions	% Of P meeting the exercise recommendation of intensity, frequency and duration	The compliance rate in the exc. group was 93.8%
Yang et al., 2011 [129]	To analyse the effect of a homebased walking exc. program on symptoms and mood in BC patients receiving chemotherapy postoperatively	N = 19 Mean age: 50.8 BC stage: I-IIIA TAIWAN	Education - Income - Ethnicity - Marital status - Employment status +	12-week: walk briskly 3 times/wk	Actual # exc. sessions completed at the prescribed intensity divided by total # exc. sessions	Adherence was approximately 77% of the prescribed exc. sessions and 100% of the prescribed exc. intensity
Zhou et al., 2019 [122]	To examine the effects of upper limb exc. and muscle relaxation training on upper limb function and HRQoL following surgery	N = 51 Mean age: 49.9 BC stage: I-III CHINA	Education + Income + Ethnicity - Marital status + Employment status +	6-M: Upper limb exercises. 10–30 min/session progression	% Of sessions completed	All patients completed with 100% compliance

Exc.: Exercise M: Month(s) Wk: Week(s) BC: BC P: Patients #: Number of.

Education was the most frequently used indicator of privilege and was found in 30 (81%) of the articles [93–122] (Table 1). Marital status was reported in 28 (76%) [93–95, 97–99, 101–109, 111–115, 122], ethnicity in 22 articles (59%) [93–95, 102, 105, 108, 110, 112, 114–116, 119, 123–128] employment status in 25 articles (68%) [93–95, 98, 101, 102, 104, 106–111, 113–115, 118, 119], and income in eight articles (22%) [95, 96, 103, 104, 114, 116, 122, 123]. Two articles [95, 114] reported five indicators, and four [124, 126, 127, 129] reported one. Ethnicity was reported in all 18 USA studies, as well as in four of the five Canadian studies, but it was not reported in the remaining studies. For the rest of the indicators, no geographical patterns were identified.

There was no uniform definition of adherence although the most common calculation was attendance rates relative to total, or weekly, prescribed, or possible, PA sessions. The mean adherence rate for these studies was 78.9% (30.4%–100%). All studies reported on patients diagnosed with BC in stages 0–III.*. Most studies reported the feasibility or effect of a PA intervention on patients undergoing radiation-, chemotherapy, or both.

The PA-interventions were designed differently in terms of PA type (e.g., walking, exercise including endurance and strength, yoga, aerobics, martial arts, dancing, cycling, qigong, balance training, Pilates), intensity (e.g., according to Ainsworth et al. [130], yoga is performed at 2.5 METs, whereas bicycling could require 4–16 METs), duration (10–60 min/session, 6 weeks–12 months/intervention) and frequency (voluntarily–5 bouts/week).

### Privileged Groups and Adherence to PA Interventions

Some articles were feasibility studies centred on adherence; others reported adherence as a sub-analysis. Six studies reported on the relationship between SES-related factors and adherence to or completion of the intervention [97, 104, 107, 109, 111, 114]. However, two of these studies reported no significant difference in participant characteristics between withdrawers and completers [111] or between those with 75% attendance and other participants [114], without specified SES-indicators. Three of these six studies indicated a positive, however weak, association between adherence to an aerobic- and strength training protocol and educational level [97, 104, 109]. One study [107] noted that employment status was associated with adherence measured as intensity, but not when adherence was assessed in terms of exercise time. No differences in adherence across educational levels were reported in this study.

### Representativeness in terms of Research Participants’ Education, Ethnicity and Marital Status

**FIGURE 2:**
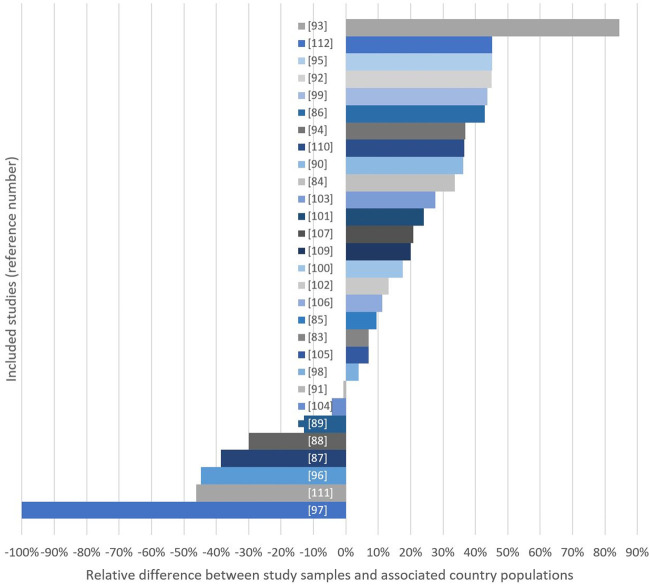
Relative differences (%) between study samples and country populations considering either mean years of education (*) or % of women with >1 year of high education in study sample. Bars extending the right side indicate studies with high-education group overrepresentation (Global, 2000–2020).

Figure 2 displays the sample-country differences in *educational* level. Eight studies had a lower proportion of individuals with higher education compared to the country population [97–99, 101, 106, 107, 114, 121], whereas 21 studies showed the opposite trend. In 11 of these studies, the proportion of participants who had tertiary education in the population was >25% lower compared to the sample [94, 96, 100, 102–105, 109, 111, 120, 122]. Four studies had a difference ≤10% [93, 95, 108, 115] (Figure 2; Table 1 in Supplementary Files S1–S3).

**FIGURE 3:**
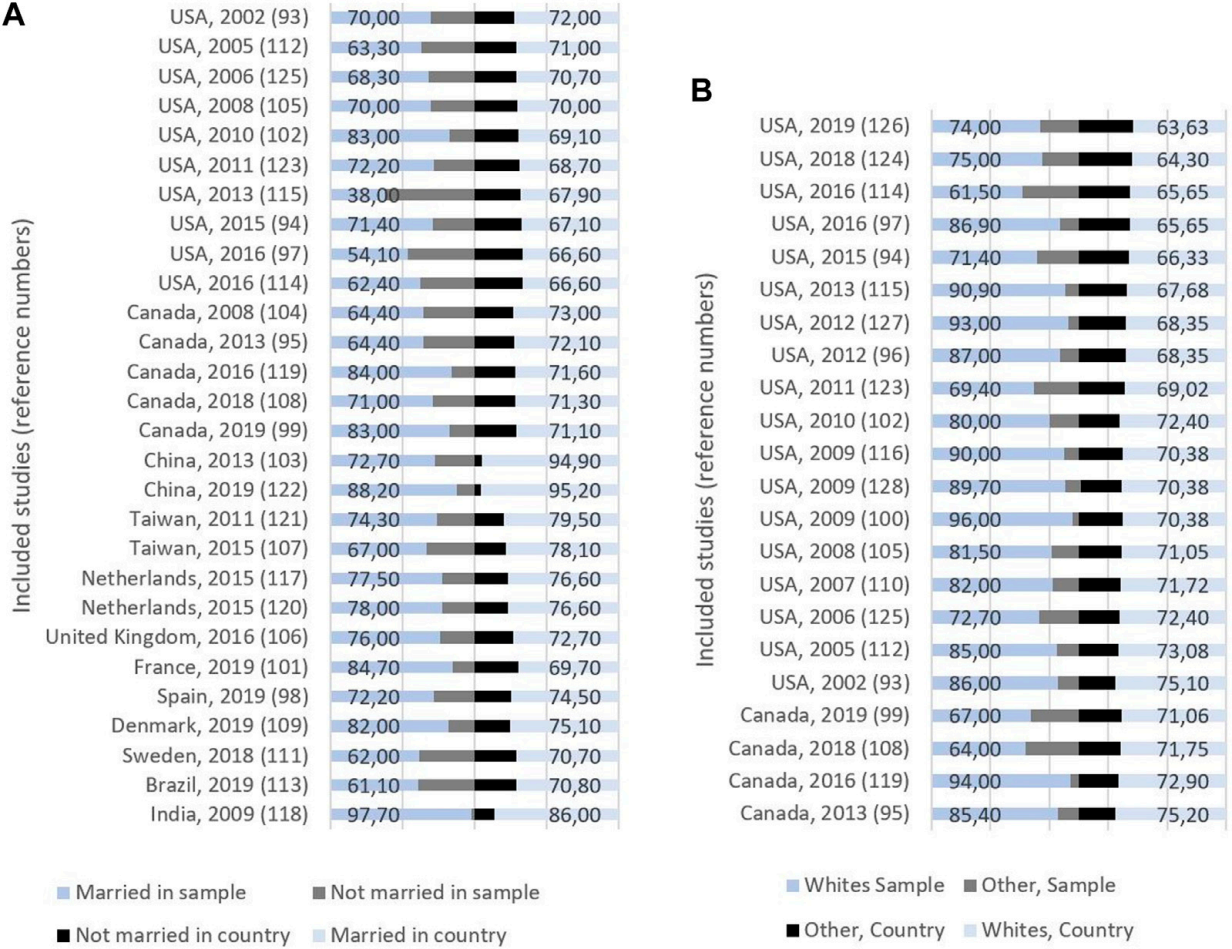
**(A)** Study origin, publication year, and proportion (%) of married and non-married women in sample (left) and country (right). **(B)** Study origin, publication year, and proportion (%) of whites and other ethnicities in sample (left) and country (right) (Global, 2000–2020).

The mean proportion of *married* women in the relevant samples was 71.9% (SD = 11.68), while the corresponding number was 73.9 (SD = 7.32) for the country populations (Figure 3; Table 2 in Supplementary File S1). Sixteen of the studies using marital status reported their results on samples with a lower proportion of married women compared to the country population [93, 95, 97, 98, 103, 104, 107, 108, 111–115, 121, 122, 125], while 11 examined samples with a higher proportion of married women compared to the country population [94, 99, 101, 102, 106, 109, 117–119, 123, 131]. One study [105] showed no difference (Tables 2, 3 in Supplementary File S1).

In the 22 studies that reported *ethnicity*, the mean proportion of whites was 81%: 77.6% Canadian studies, and 81.8% American studies (Figure 3; Table 3 in Supplementary File S1). Three of these studies reported their results on samples with a lower proportion of whites compared with the country population, while the remaining 19 studies had samples with a higher proportion of whites. There was a difference between samples and countries of 10%–25% in 15 of these studies (Table 3 in Supplementary File S1).

### Representativeness and Adherence

There was no clear relationship between the registered representativeness and the associations between indicators of SES and adherence. Three of the six mentioned studies reporting such associations [97, 107, 114] had samples with a lower proportion of high-SES BC patients compared to their country populations, while the other three [104, 109, 111] had samples with a higher proportion of high-SES patients.

## Discussion

To our knowledge, this is the first systematic review to report the social representativeness of RCTs examining the effects of PA on BC-related outcomes. In the included dataset, there was a preponderance of studies overrepresenting groups associated with higher privilege, supporting previous results indicating social bias [132]. Misrepresentations were found across different indicators of privilege, likely because different indicators tend to interrelate [133]. Our findings coincide with earlier observations that low-SES patients are less inclined to participate in research than high-SES patients [62, 63], as well as with previous findings of obstacles to recruiting less privileged populations to cancer RCTs [64].

The most pronounced findings were based on studies that reported participants’ education. However, in six studies that did not record education but rather recorded ethnicity/race, as in the total dataset, there was a preponderance of white participants. In agreement with previous reports [62, 132, 134], this was interpreted as recruitment bias, although these analyses were based on less accurate data compared to the analyses on education. All 22 studies reporting ethnicity were from the USA and Canada, where the ethnic distribution varies over time and space, particularly in the United States [91]. Although education and income relate to ethnicity [135], our results on the representativeness of ethnic groups are unclear due to inaccurate data and increased confusion among the populations regarding ethnic categories. In general, it may appear inappropriate to use the category white as an exclusive indicator of social privilege in global comparative analyses such as the current study. Nonetheless, in the overall discussion of representativeness in RCTs, research favouring certain ethnicities over others should not be supported.

Representativeness was further assessed by marital status, and the mean difference between the study samples and the associated country populations was small. However, the differences ranged from −78% to 17.7%, and only 9 studies differed less than 5% from the country population. This means that although marital status is considered a measure of whether people have the social and financial benefits of being coupled, it could affect the possibility of being included in a PA trial.

What is considered an acceptable level of representativeness in RCT samples is difficult to decide. Recruitment issues may lead to sample biases, as patient populations often differ across social groups due to their diagnoses or hospital characteristics. However, the body of articles from the current dataset that employed education as the SES-indicator included eight studies with **(**“negative” differences from the country population and 22 studies with “positive” differences (including 11 samples with >25% difference from the country population), which are significant findings. The fact that studies with a negative deviation from the country’s corresponding distribution in this subgroup were published after 2015 could be an effect of the CONSORT guidelines [70] and the Belmont report [136], which emphasised the implications of selection biases. However, our screening and analyses clearly show that there is still a potential for improvement among researchers in their attention to social distribution in samples.

Our results may have been more valid if baseline characteristics were compared with a real-world patient population and a patient sample (see [4]). However, such analyses require that third-party researchers also have access to exact data about the patient populations within each region, at the precise time of the studies, in addition to which patients are eligible according to each of the RCT criteria. The primary aim of the present study was to revive the question of external validity and feasibility from a perspective of social health inequalities in PA research. Therefore, status distribution in the respective age groups within the country populations served as an acceptable proxy for the distribution in the patient population.

Another objection may be that, for a patient to be considered eligible for a PA RCT, their cancer must be at a stage compatible with physical exertion and that more advanced BCs are associated with lower privilege [137]. Hence, RCTs with exercise often include patients with higher SES (education) because they have less advanced BC. However, this claim confirms the importance of putting focus on representativeness in RCTs targeting an already socially skewed patient population; it is important to be even more aware of sample representativeness to avoid excessive bias by favouring the already privileged. Likewise, employing already skewed patient populations as a reference could promote further social inequalities in health. Nonetheless, if the reference ratio for assessing representativeness was 1.22 [17], it would be possible to accept 22% more high-education patients before claiming misrepresentation of less privileged groups. However, according to our results, based on patients’ educational levels, approximately 45% of the included studies would still be considered biased in favour of the most privileged patients.

### SES and PA Intervention Adherence

A proper body of articles with analyses of the association between SES-related characteristics and adherence was expected, and also that these articles would provide a basis for a synthesised examination of this relationship. However, the fact that only 6 studies investigated this association suggests that this question is perceived to be of little relevance in the field of BC and PA research. A previous review on the representativeness of RCTs reported no socioeconomic misrepresentation in oncology studies [4] however, our interpretation is supported by the fact that the review included only articles that provided a representativeness analysis, which the oncology articles had omitted in the case of SES.

No clear trend of associations between SES-related factors and adherence was seen across the six aforementioned studies, and the small number of studies and the differences between them hampered a clear conclusion. Previous studies have argued that poor representativeness may explain a lack of associations between SES and adherence rates [138, 139]. In the current data set, there was no clear association between representativeness and SES-adherence associations. However, the small number of relevant articles formed an overly scarce base for reliable and conclusive analyses of this research question.

A tendency for studies that found a positive relationship between SES-related factors and adherence to report on longer interventions and to calculate adherence in terms of rates of attendance was observed. Three studies reported that the adherence rates decreased over time [97, 104, 107]. Previous results support the interpretation that time is a barrier to participation in PA interventions [53, 140]. Strazdin et al. have substantiated how availability of time may have a larger impact on low-SES women in general [141, 142]. Hence, the limited availability of time over a prolonged period may partly explain why less privileged patients have poorer adherence in such trials than their privileged counterparts.

Reasons for refusing to participate, or reasons for withdrawing, were not registered in our study. We should not ignore the fact that researchers experience real obstacles in recruiting of less privileged patients, so non-representative samples should not be seen as unwillingness or lack of effort to include the less privileged. There are reasons to believe that reasons for refusal to participate in the articles included coincide with the most common barriers previously reported [10, 64, 131]*.* However, although participation is voluntary and individual motivation or barriers are relevant, the adherence rates and the current social group distribution of patients in RCTs with PA may also be affected by the intervention design and the inclusion criteria defined by the researchers of each individual study [64].

All the included studies reported LTPA interventions. Considering that low-SES groups engage less in LTPA [47], it is reasonable to expect that low-SES patients were less inclined to participate in, and complete, such RCTs. Hence, a preponderance of RCTs employing exercise interventions could be perceived as being in favour of privileged patients. The searches of the current study did not, however, include search terms covering other PA domains and may thereby have strengthened the impression that LTPA is the prevailing protocol in RCTs of PA in BC patients. However, although many patients return to work during their first-year post diagnosis, RCTs designed for other PA-domains would not be verifiable as the patients’ level of PA would differ too much to control. In addition, the control group would have to be inactive within these PA-domains, which would be a requirement almost impossible to implement. The probability that many published RCTs of other types of PA exist, thus altering our results, is therefore small.

### Consequences of Enhanced Selection Bias in Favour of Privileged Patients

Provided that our results reflect reality as they are based on synthesised data from a large set of systematically selected articles, social equity is a challenge to PA trials in BC patients. A stepwise, albeit unintended, social exclusion process causes attrition bias and a successive decrease in external validity, (i.e., the results are even less valid for individuals in other social groups than those who have completed the intervention when the intervention is finalised) [65]. Thus, PA-treatment is also less adaptable to patients in the same groups as those who withdraw. Drawing on the fundamental cause theory [143], these mechanisms show how affluent people could benefit more, in this case, from PA research, because studies serve the interests of these groups more than they care for all SES-groups. In addition, insignificant results from sub-analyses of social group-differences may be misinterpreted because the distribution of social groups in the samples does not mirror real life. This may disguise the possible fact that although all patients, by policy, should have equal access to health services, PA treatment interventions may not be suitable for all. An “inverse PA research law” analogous to the “inverse healthcare law” [144] describes how PA research interventions, initiated with the intention to treat a patient population, regardless of social status, nevertheless attend to privileged groups more than the less privileged who initially are prone to poorer health and thus more entitled to clinical research.

### Strengths and Limitations

The major strength of this research is the thorough and systematic search of eligible studies, including traditional, and both BCB and FCB searches. To avoid our searches and not be included, an article would have to be published in a journal that is not indexed in any of the chosen databases, not be identified by the search terms, not be included in either the reference lists of the articles included from the descriptive searches or not have cited the articles included from the descriptive searches. It is possible that some articles were lost. However, it is less likely that any articles not included would be systematically more representative compared with the articles included.

The sample sizes in one-third of the studies were <50, seemingly decreasing the validity of our conclusions. However, rather than being a shortcoming of our study, the limitation is applicable to each RCT because the small samples, among other factors, hamper representativeness analyses such as ours. Hence, our analyses were based on the publication reality, whatever flawed original data materials.

SES and social privilege classifications vary across cultures, research fields, and public statistics, and they may cause imprecise standards for comparisons between studies of different origins. Nevertheless, our results present the big picture of misrepresentation across social groups with a precision sufficient to boost the debate within the scientific community.

### Conclusion

Less educated, unmarried and non--white individuals may be underrepresented in BC PA RCTs, and SES-adherence associations in such trials are inconclusive. Unintentional social misrepresentation may create disguised inequity, warranting revived attention to this issue.

The current study provides a departure point for intensified attention to representativeness in RCTs. It should act as motivation both for seeking improved external validity, and for reconsidering whether LTPA in BC treatment is suitable for all.
