# Supplementary material for: Social Representativeness and Intervention Adherence—A Systematic Review of Clinical Physical Activity Trials in Breast Cancer Patients
Source: Int J Public Health. 2024 May 9;69:1607002. doi: 10.3389/ijph.2024.1607002 (PMC11111874; doi:10.3389/ijph.2024.1607002)
Supplement: Supplementary file 1 [file DataSheet3.pdf]

3.Enrollment Rates of School –Total Net Enrollment Rate

Unit：％

	Total			Compulsory Education			Primary			Primary (de jure population)			Lower secondary			Upper secondary			Secondary			Tertiary			Preschool		
	Average	M.	F.	Average	M.	F.	Average	M.	F.	Average	M.	F.	Average	M.	F.	Average	M.	F.	Average	M.	F.	Average	M.	F.	Average	M.	F.
76'	67.57	69.51	65.52	92.63	94.13	91.05	97.54	97.49	97.59	…	…	…	77.33	81.60	72.82	43.17	46.08	40.11	65.73	69.53	61.71	9.97	11.15	8.73	10.45	10.81	10.06
81'	69.52	69.75	69.28	95.13	95.44	94.80	97.59	97.54	97.64	…	…	…	84.41	85.26	83.52	52.58	52.03	53.17	72.96	73.19	72.72	11.47	12.15	10.75	14.87	15.32	14.39
86'	74.42	73.99	74.86	95.95	95.99	95.90	96.75	96.73	96.77	…	…	…	89.12	89.18	89.06	66.45	64.49	68.52	81.50	80.52	82.53	14.24	14.47	14.00	19.98	20.41	19.52
87'	75.34	74.79	75.93	96.09	96.15	96.02	96.97	96.96	96.97	…	…	…	89.02	89.27	88.76	68.43	66.05	70.94	82.39	81.13	83.73	14.82	14.90	14.74	21.77	22.10	21.41
88'	76.35	75.60	77.15	96.40	96.40	96.40	97.96	97.94	97.99	…	…	…	87.93	88.00	87.86	70.42	67.02	74.00	82.88	81.25	84.61	15.95	15.86	16.04	22.80	23.20	22.38
89'	76.55	75.64	77.52	96.77	96.64	96.90	97.74	97.74	97.74	…	…	…	89.82	89.32	90.35	70.80	67.38	74.38	83.88	81.95	85.91	17.18	16.67	17.72	23.75	24.02	23.48
90'	77.97	77.06	78.92	97.12	97.15	97.09	98.04	98.21	97.87	…	…	…	90.31	90.05	90.59	73.01	69.36	76.85	85.45	83.64	87.35	19.36	18.33	20.44	23.55	23.84	23.23
91'	78.74	77.98	79.55	97.97	97.95	97.98	98.70	98.75	98.64	…	…	…	91.70	91.49	91.93	72.93	69.34	76.71	86.17	84.40	88.05	20.98	20.22	21.78	23.30	23.47	23.12
92'	79.35	78.68	80.07	97.60	97.45	97.76	98.92	98.93	98.91	…	…	…	90.69	90.23	91.17	75.49	72.31	78.84	86.46	84.76	88.26	23.47	23.04	23.92	22.99	23.08	22.88
93'	79.79	79.15	80.47	98.12	98.02	98.23	99.31	99.32	99.30	…	…	…	91.63	91.25	92.02	77.28	74.15	80.59	87.43	85.80	89.15	25.61	24.99	26.26	23.06	23.24	22.86
94'	79.11	78.21	80.07	97.47	97.26	97.68	98.36	98.34	98.38	…	…	…	92.63	92.02	93.28	78.57	74.89	82.47	88.59	86.59	90.71	26.26	24.99	27.60	23.40	23.51	23.27
95'	79.38	78.39	80.43	98.36	98.23	98.49	99.06	99.01	99.10	…	…	…	94.14	93.83	94.47	79.15	75.84	82.65	88.84	87.03	90.75	27.79	25.88	29.78	23.44	23.71	23.14
96'	78.95	77.90	80.07	98.23	98.04	98.43	99.02	98.88	99.17	…	…	…	94.27	93.97	94.58	80.30	77.44	83.34	89.57	87.93	91.31	29.07	26.88	31.37	23.45	23.78	23.08
97'	79.26	78.14	80.45	98.38	98.24	98.54	98.62	98.56	98.69	…	…	…	95.60	95.29	95.93	81.74	78.90	84.75	90.79	89.23	92.45	31.09	28.35	33.97	23.34	23.53	23.13
98'	80.04	78.91	81.25	98.15	97.97	98.36	97.78	97.72	97.83	…	…	…	96.15	95.73	96.60	83.34	81.19	85.63	91.68	90.31	93.13	33.32	30.19	36.61	23.62	24.00	23.22
99'	80.40	79.29	81.58	98.24	98.14	98.36	97.81	97.84	97.78	…	…	…	96.52	96.17	96.89	84.95	82.80	87.24	92.56	91.29	93.91	35.43	32.14	38.90	23.27	23.49	23.03
00'	81.23	80.17	82.36	98.00	97.96	98.03	98.78	98.77	98.79	…	…	…	93.96	93.83	94.11	87.08	84.90	89.39	92.19	90.96	93.49	38.70	35.47	42.11	24.47	24.60	24.33
01'	82.29	81.18	83.48	97.32	97.29	97.35	98.19	98.20	98.16	…	…	…	93.53	93.42	93.65	88.21	86.00	90.56	92.92	91.83	94.07	42.51	38.98	46.23	22.94	22.73	23.18
02'	83.44	82.35	84.61	97.15	97.13	97.16	98.04	98.09	97.99	…	…	…	93.47	93.27	93.69	89.32	87.54	91.23	93.74	92.84	94.72	45.68	42.14	49.41	25.74	25.79	25.68
03'	84.63	83.57	85.78	96.74	96.77	96.70	97.30	97.34	97.26	…	…	…	92.41	92.41	92.40	87.63	86.13	89.24	93.83	93.03	94.69	49.05	45.33	52.99	25.31	25.47	25.14
04'	86.55	85.59	87.60	97.68	97.69	97.67	98.23	98.32	98.13	…	…	…	93.00	92.91	93.09	88.44	87.08	89.90	93.63	92.94	94.39	53.20	49.58	57.04	25.97	26.11	25.82
05'	87.71	86.81	88.69	98.68	98.69	98.67	98.46	98.49	98.44	…	…	…	96.51	96.50	96.51	88.53	87.32	89.85	93.63	93.05	94.26	57.42	54.00	61.06	27.68	27.81	27.53
06'	88.55	87.74	89.43	98.11	98.17	98.05	97.77	97.83	97.71	…	…	…	96.65	96.72	96.57	91.31	90.15	92.57	94.91	94.42	95.43	59.83	56.70	63.16	27.57	27.57	27.57
07'	89.26	88.49	90.09	97.84	97.90	97.78	97.79	97.87	97.69	…	…	…	96.86	96.90	96.81	90.72	89.94	91.59	94.56	94.23	94.92	61.41	58.33	64.71	27.70	27.64	27.77
08'	89.36	88.54	90.25	98.00	98.05	97.94	98.02	98.09	97.95	...	...	...	97.95	97.99	97.91	92.62	91.91	93.41	95.29	94.95	95.67	68.74	65.89	71.80	27.90	27.91	27.88
09'	89.44	88.53	90.43	98.17	98.24	98.09	98.14	98.23	98.04	99.24	99.28	99.20	98.21	98.27	98.16	92.97	92.17	93.85	95.60	95.22	96.01	69.26	66.24	72.53	28.50	28.62	28.37
10'	89.55	88.57	90.62	98.09	98.17	98.00	98.06	98.15	97.97	99.21	99.24	99.17	98.13	98.20	98.05	93.46	92.80	94.17	95.76	95.46	96.08	70.13	66.85	73.70	29.49	29.63	29.34
11'	89.76	88.71	90.91	98.04	98.12	97.96	97.98	98.08	97.87	99.25	99.40	99.08	98.15	98.19	98.10	93.62	92.91	94.39	95.79	95.44	96.17	71.62	68.21	75.32	30.93	31.05	30.80
12'	89.85	88.72	91.08	97.99	98.08	97.90	97.88	98.00	97.75	99.30	99.42	99.18	98.18	98.21	98.14	93.68	92.86	94.56	95.80	95.38	96.24	72.63	69.10	76.47	58.59	59.13	58.00
13'	89.79	88.56	91.14	97.94	98.00	97.86	97.78	97.87	97.69	99.45	99.56	99.32	98.18	98.22	98.14	93.83	93.08	94.64	95.90	95.52	96.30	73.09	69.19	77.30	58.30	58.89	57.67
14'	89.71	88.34	91.19	97.86	97.94	97.77	97.68	97.77	97.59	99.46	99.58	99.34	98.13	98.22	98.04	94.10	93.40	94.85	96.03	95.71	96.38	73.41	69.10	78.05	56.51	57.12	55.85
15'	89.53	88.03	91.16	97.79	97.87	97.71	97.56	97.63	97.49	99.53	99.59	99.46	98.17	98.28	98.06	94.25	93.60	94.97	96.09	95.80	96.42	73.49	68.86	78.49	57.23	57.79	56.63
16'	89.39	87.80	91.13	97.65	97.72	97.57	97.43	97.51	97.34	99.54	99.62	99.45	98.03	98.09	97.97	94.46	93.76	95.23	96.08	95.72	96.47	73.49	68.65	78.71	59.20	59.80	58.56
17'	89.24	87.64	90.99	97.51	97.58	97.44	97.27	97.34	97.20	99.58	99.64	99.51	97.94	98.01	97.86	94.28	93.60	95.01	95.92	95.59	96.29	73.25	68.38	78.52	60.59	61.03	60.12
18'	89.21	87.61	90.96	97.35	97.40	97.31	97.14	97.19	97.08	99.52	99.57	99.47	97.75	97.78	97.72	94.17	93.52	94.88	95.80	95.47	96.17	73.38	68.58	78.60	63.22	63.65	62.74
19'	89.11	87.55	90.81	97.19	97.22	97.15	97.02	97.07	96.97	99.47	99.52	99.42	97.51	97.51	97.50	94.25	93.50	95.08	95.78	95.39	96.22	72.98	68.34	78.02	67.37	67.86	66.84
20'	89.83	88.31	91.49	97.42	97.45	97.40	97.34	97.38	97.30	99.69	99.73	99.65	97.59	97.57	97.61	94.40	93.67	95.21	95.93	95.54	96.36	74.41	69.86	79.36	71.07	71.58	70.53
21'	90.66	89.17	92.27	97.76	97.75	97.77	97.69	97.69	97.68	99.79	99.79	99.78	97.90	97.86	97.95	94.76	93.99	95.60	96.28	95.86	96.75	75.84	71.41	80.66	74.60	75.03	74.14

Note：
1. In accordance with UNESCO's definition, Net Enrollment Rate and the data from school year 2008 above were revised into Total Net Enrollment Rate, Total Net Enrollment Rate= total number of students of the official age group at all levels of education ÷ total number of corresponding population for a given level of education × 100%. Net Enrollment Rate = total number of students of the official age group for a given level of education ÷ total number of corresponding population for a given level of education × 100%.Net Enrollment Rate in tertiary education includes religious colleges' student.
2. Since the "Early Childhood Education and Care Act" was fulfilled in 2012, the kindergartens and the child-care centers were reformed to the "Preschools" from the SY 2012-2013.
3. Net enrollment rate of students in the primary school aged 6 to 11 (de jure population) = number of school-age students in the primary school aged 6 to 11 ÷ number of permanent school-age children aged 6 to 11.

Source: Ministry of Education RoC, (Taiwan) Enrollment Rates of School - Total Net Enrollment Rates: Ministry of Education 2022 [updated 22-05-04; cited 2023 07 Nov 2023]. Available from: https://stats.moe.gov.tw/files/ebook/indicators/13.pdf
