# Supplementary material for: Social Representativeness and Intervention Adherence—A Systematic Review of Clinical Physical Activity Trials in Breast Cancer Patients
Source: Int J Public Health. 2024 May 9;69:1607002. doi: 10.3389/ijph.2024.1607002 (PMC11111874; doi:10.3389/ijph.2024.1607002)
Supplement: Supplementary file 2 [file DataSheet1.docx]

Table 1 Mean years of education or % with >1 years of high education in samples and country population. (Global, 2000-2020).

| **Study origin,**  **Year of publication** | **Basis for the calculation of**  **>1 years of high education** | **Mean years of education or**  **% with >1 years of high education**  **in study sample** | **Mean years of education or**  **% with >1 years of high education**  **in country population** | **Difference between study sample and country population**  **relative to sample (%)** |
| --- | --- | --- | --- | --- |
| **USA, 2002 [1]** | Education, mean years | 14.00 years | 13.00 years ^a^ | 7.10 |
| **USA, 2005 [2]** | Education, mean years | 15.10 years | 13.10 years ^a^ | 13.20 |
| **USA, 2007 [3]** | Years of education | 16.00 years | 13.20 years ^a^ | 17.50 |
| **USA, 2009 [4]** | Education years | 15.00 years | 13.30 years ^a^ | 11.30 |
| **USA, 2008 [5]** | At least some college education | 78.00 % | 42.80% **^b^** | 45.10 |
| **USA, 2009 [6]** | College degree or higher | 68.00 % | 43.40% **^b^** | 36.20 |
| **USA, 2010 [7]** | Some college or higher education | 80.00 % | 44.00% **^b^** | 45.00 |
| **USA, 2012 [8]** | Some college or college graduate | 79.00% | 45.10% **^b^** | 42.90 |
| **USA, 2013 [9]** | Bachelor, Master, or PhD degree | 49.90% | 46.40% **^b^** | 7.00 |
| **USA, 2015 [10]** | College or post college | 71.50% | 47.40% **^b^** | 33.70 |
| **USA, 2016 [11]** | College degree or graduate degree | > 35.00% | 48.50% **^b^** | -38.60 |
| **USA, 2016 [12]** | College or higher | 46.50% | 48.50% **^b^** | -4.30 |
| **Canada, 2008 [13]** | University or graduate school | 83.80% | 52.90% **^b^** | 36.90 |
| **Canada, 2013 [14]** | Completed university | 64.80% | 58.70% **^b^** | 9.40 |
| **Canada, 2016 [15]** | ≥College/university degree | 78.00% | 62.40% **^b^** | 20.00 |
| **Canada, 2018 [16]** | Some univ., Bachelor, or >Bachelor | 67.00% | 64.40% **^b^** | 3.90 |
| **Canada, 2019 [17]** | ≥Bachelor’s degree | 58.00% | 65.50% **^b^** | -12.90 |
| **Brazil, 2019 [18]** | Higher education | 29.40% | 21.30% **^b^** | 27.60 |
| **France, 2019 [19]** | ≥2 years at university | 40.30% | 40.60% **^b^** | -0.70 |
| **Netherlands, 2015 [20]** | High | >46.10% | 36.50% **^b^** | 20.80 |
| **Netherlands, 2015 [21]** | College/university | 57.50% | 36.50% **^b^** | 36.50 |
| **Spain, 2019 [22]** | High | >31.80% | 41.30% **^b^** | -29.90 |
| **UK, 2016 [23]** | Degree or higher degree | 32.00% | 46.30% **^b^** | -44.70 |
| **Denmark, 2019 [24]** | Long | 81.50% | 45.90% **^b^** | 43.70 |
| **Sweden, 2018 [25]** | Tertiary | 66.20% | 50.30% **^b^** | 24.00 |
| **India, 2009 [26]** | High school education (inclusion) | >0.00% | 7.80% **^b^** * | Not assessed due to inclusion criteria |
| **Taiwan, 2015 [27]** | ≥College | 20.60% | 78.49% ^c^ | -281.00 |
| **Taiwan, 2011 [28]** | College or graduated | 51.50% | 75.32% ^c^ | -46.25 |
| **China, 2013 [29]** | Some college or higher | 55.00% | 8.60% **^b^**** | 84.40 |
| **China, 2019 [30]** | Tertiary:15.7% | 15.70% | 8.60% **^b^**** | 45.20 |

^a^ Based on: UNESCO via World Bank [31] [32] Statistics retrieved from the year in the database closest to the study's publication year

^b^ Based on: OECD’s Education Statistics [33]

^b^*2011-data due to missing data from earlier periods

**^b^**** Latest available data (2010)

^c^ Based on: Ministry of Education, Republic of China (Taiwan). Educational statistics [34]

Table 2 Married women in samples and country (45-49 years) at publication year. Differences relative to sample (%). (Global, 2000-2020).

| **Study origin,**  **Year of publication** | **Sample**  **(%)** | **Country^a^**  **(%)** | **Relative difference**  **(%)** |
| --- | --- | --- | --- |
| **USA, 2002 [1]** | 70.00 | 72.00 | -2.85 |
| **USA, 2005 [2]** | 63.30 | 71.00 | -12.16 |
| **USA, 2006 [35]** | 68.30 | 70.70 | -3.51 |
| **USA, 2008 [5]** | 70.00 | 70.00 | 0.00 |
| **USA, 2010 [7]** | 83.00 | 69.10 | 16.74 |
| **USA, 2011 [36]** | 72.20 | 68.70 | 4.84 |
| **USA, 2013 [9]** | 38.00 | 67.90 | -78.68 |
| **USA, 2015 [10]** | 71.40 | 67.10 | 6.02 |
| **USA, 2016 [11]** | 54.10 | 66.60 | -23.10 |
| **USA, 2016 [12]** | 62.40 | 66.60 | -6.73 |
| **Canada, 2008 [13]** | 64.40 | 73.00 | -13.35 |
| **Canada, 2013 [14]** | 64.40 | 72.10 | -11.95 |
| **Canada, 2016 [15]** | 84.00 | 71.60 | 14.76 |
| **Canada, 2018 [16]** | 71.00 | 71.30 | -0.42 |
| **Canada, 2019 [17]** | 83.00 | 71.10 | 14.33 |
| **China, 2013 [29]** | 72.70 | 94.90 | -30.53 |
| **China, 2019 [30]** | 88.20 | 95.20 | -7.93 |
| **Taiwan, 2011 [28]** | 74.30 | 79.50 | -6.99 |
| **Taiwan, 2015 [27]** | 67.00 | 78.10 | -16.56 |
| **Netherlands, 2015 [20]** | 77.50 | 76.60 | 1.16 |
| **Netherlands, 2015 [21]** | 78.00 | 76.60 | 1.79 |
| **United Kingdom, 2016 [23]** | 76.00 | 72.70 | 4.34 |
| **France, 2019 [19]** | 84.70 | 69.70 | 17.70 |
| **Spain, 2019 [22]** | 72.20 | 74.50 | -3.18 |
| **Denmark, 2019 [24]** | 82.00 | 75.10 | 8.40 |
| **Sweden, 2018 [25]** | 62.00 | 70.70 | -14.03 |
| **Brazil, 2019 [18]** | 61.10 | 70.80 | -15.87 |
| **India, 2009 [26]** | 97.70 | 86.00 | 11.97 |

^a^ Based on: United Nations World Marriage Data (age 45-49) [37]

Table 3 Whites in samples, and country population estimated through interpolation. Differences relative to sample (%). (Global, 2000-2020).

| **Study origin,**  **Year of publication** | **Sample**  **(%)** | **Country^a^**  **(%)** | **Relative difference**  **(%)** |
| --- | --- | --- | --- |
| **Canada, 2013 [14]** | 85.40 | 75.20 | 11.94 |
| **Canada, 2016 [15]** | 94.00 | 72.90 | 22.45 |
| **Canada, 2018 [16]** | 64.00 | 71.75 | -12.11 |
| **Canada, 2019 [17]** | 67.00 | 71.06 | -6.06 |
| **USA, 2002 [1]** | 86.00 | 75.10 | 12.67 |
| **USA, 2005 [2]** | 85.00 | 73.08 | 14.03 |
| **USA, 2006 [35]** | 72.70 | 72.40 | 0.41 |
| **USA, 2007 [3]** | 82.00 | 71.72 | 12.53 |
| **USA, 2008 [5]** | 81.50 | 71.05 | 12.82 |
| **USA, 2009 [6]** | 96.00 | 70.38 | 26.69 |
| **USA, 2009 [38]** | 89.70 | 70.38 | 21.54 |
| **USA, 2009 [4]** | 90.00 | 70.38 | 21.81 |
| **USA, 2010 [7]** | 80.00 | 72.40 | 9.50 |
| **USA, 2011 [36]** | 69.40 | 69.02 | 0.54 |
| **USA, 2012 [8]** | 87.00 | 68.35 | 21.44 |
| **USA, 2012 [39]** | 93.00 | 68.35 | 26.51 |
| **USA, 2013 [9]** | 90.90 | 67.68 | 25.55 |
| **USA, 2015 [10]** | 71.40 | 66.33 | 7.11 |
| **USA, 2016 [11]** | 86.90 | 65.65 | 24.45 |
| **USA, 2016 [12]** | 61.50 | 65.65 | -6.75 |
| **USA, 2018 [40]** | 75.00 | 64.30 | 14.27 |
| **USA, 2019 [41]** | 74.00 | 63.63 | 14.01 |

^a^ Figures for intercensal years for the Canadian studies were calculated by interpolating between the census years 2011, 2016 and 2021 (Statistics Canada) [42-44] , using the ‘White’+‘Whites and other’ categories, following Statistics Canada [45]. For the USA studies, figures for intercensal years were calculated by interpolating between the census years 2000, 2010 and 2020 (US census Bureau, Race and Ethnicity in the United States) [46,47], using the ‘White alone’ category.

**References:**

1. Pickett M, Mock V, Ropka ME, et al. Adherence to moderate-intensity exercise during breast cancer therapy. Cancer Pract. 2002 Nov-Dec;10(6):284-92.

2. Mock V, Frangakis C, Davidson NE, et al. Exercise manages fatigue during breast cancer treatment: A randomized controlled trial. Psycho-Oncology. 2005 Jun;14(6):464-477.

3. Matthews CE, Wilcox S, Hanby CL, et al. Evaluation of a 12-week home-based walking intervention for breast cancer survivors. Supportive Care in Cancer. 2007 Feb;15(2):203-211.

4. Rogers LQ, Hopkins-Price P, Vicari S, et al. A randomized trial to increase physical activity in breast cancer survivors. Med Sci Sports Exerc. 2009 Apr;41(4):935-46.

5. Demark-Wahnefried W, Case LD, Blackwell K, et al. Results of a diet/exercise feasibility trial to prevent adverse body composition change in breast cancer patients on adjuvant chemotherapy. Clin Breast Cancer. 2008 Feb;8(1):70-9.

6. Cadmus LA, Salovey P, Yu H, et al. Exercise and quality of life during and after treatment for breast cancer: results of two randomized controlled trials. Psycho-Oncology. 2009 Apr;18(4):343-352.

7. Chandwani KD, Thornton B, Perkins GH, et al. Yoga improves quality of life and benefit finding in women undergoing radiotherapy for breast cancer. Journal of the Society for Integrative Oncology. 2010;8(2).

8. Anderson RT, Kimmick GG, McCoy TP, et al. A randomized trial of exercise on well-being and function following breast cancer surgery: the RESTORE trial. Journal of Cancer Survivorship-Research and Practice. 2012 Jun;6(2):172-181.

9. Reis D, Walsh ME, Young-McCaughan S, et al., editors. Effects of Nia exercise in women receiving radiation therapy for breast cancer. Oncology Nursing Forum; 2013.

10. Al-Majid S, Wilson LD, Rakovski C, et al. Effects of exercise on biobehavioral outcomes of fatigue during cancer treatment: results of a feasibility study. Biological Research for Nursing. 2015;17(1):40-48.

11. Arem H, Sorkin M, Cartmel B, et al. Exercise adherence in a randomized trial of exercise on aromatase inhibitor arthralgias in breast cancer survivors: the Hormones and Physical Exercise (HOPE) study. Journal of Cancer Survivorship. 2016 Aug;10(4):654-62.

12. Ratcliff CG, Milbury K, Chandwani KD, et al. Examining mediators and moderators of yoga for women with breast cancer undergoing radiotherapy. Integrative Cancer Therapies. 2016;15(3):250-262.

13. Courneya KS, Segal RJ, Gelmon K. Predictors of supervised exercise adherence during breast cancer chemotherapy. Med Sci Sports Exerc. 2008;40.

14. Courneya KS, McKenzie DC, Mackey JR, et al. Effects of exercise dose and type during breast cancer chemotherapy: multicenter randomized trial. J Natl Cancer I. 2013;105(23):1821-1832.

15. Vallance JK, Friedenreich CM, Lavallee CM, et al. Exploring the Feasibility of a Broad-Reach Physical Activity Behavior Change Intervention for Women Receiving Chemotherapy for Breast Cancer: A Randomized Trial. Cancer Epidemiol Biomarkers Prev. 2016 Feb;25(2):391-8.

16. Kirkham AA, Van Patten CL, Gelmon KA, et al. Effectiveness of Oncologist-Referred Exercise and Healthy Eating Programming as a Part of Supportive Adjuvant Care for Early Breast Cancer. Oncologist. 2018 Jan;23(1):105-115.

17. Bland KA, Kirkham AA, Bovard J, et al. Effect of exercise on taxane chemotherapy–induced peripheral neuropathy in women with breast cancer: a randomized controlled trial. Clinical breast cancer. 2019;19(6):411-422.

18. Paulo TR, Rossi FE, Viezel J, et al. The impact of an exercise program on quality of life in older breast cancer survivors undergoing aromatase inhibitor therapy: A randomized controlled trial. Health Qual Life Out. 2019;17(1):1-12.

19. Carayol M, Ninot G, Senesse P, et al. Short-and long-term impact of adapted physical activity and diet counseling during adjuvant breast cancer therapy: the “APAD1” randomized controlled trial. BMC cancer. 2019;19(1):1-20.

20. Travier N, Velthuis MJ, Bisschop CNS, et al. Effects of an 18-week exercise programme started early during breast cancer treatment: a randomised controlled trial. Bmc Medicine. 2015 Jun 8;13.

21. van Waart H, Stuiver MM, van Harten WH, et al. Effect of Low-Intensity Physical Activity and Moderate- to High-Intensity Physical Exercise During Adjuvant Chemotherapy on Physical Fitness, Fatigue, and Chemotherapy Completion Rates: Results of the PACES Randomized Clinical Trial. J Clin Oncol. 2015 Jun 10;33(17):1918-27.

22. Ariza-Garcia A, Lozano-Lozano M, Galiano-Castillo N, et al. A web-based exercise system (e-CuidateChemo) to counter the side effects of chemotherapy in patients with breast cancer: randomized controlled trial. J Med Internet Res. 2019;21(7):e14418.

23. Gokal K, Wallis D, Ahmed S, et al. Effects of a self-managed home-based walking intervention on psychosocial health outcomes for breast cancer patients receiving chemotherapy: a randomised controlled trial. Support Care Cancer. 2016 Mar;24(3):1139-66.

24. Lund LW, Ammitzbøll G, Hansen DG, et al. Adherence to a long-term progressive resistance training program, combining supervised and home-based exercise for breast cancer patients during adjuvant treatment. Acta Oncologica. 2019;58(5):650-657.

25. Mijwel S, Backman M, Bolam KA, et al. Adding high-intensity interval training to conventional training modalities: optimizing health-related outcomes during chemotherapy for breast cancer: the OptiTrain randomized controlled trial. Breast Cancer Res Tr. 2018 Feb;168(1):79-93.

26. Vadiraja SH, Rao MR, Nagendra RH, et al. Effects of yoga on symptom management in breast cancer patients: A randomized controlled trial. Int J Yoga. 2009 Jul;2(2):73-9.

27. Huang HP, Wen FH, Tsai JC, et al. Adherence to prescribed exercise time and intensity declines as the exercise program proceeds: findings from women under treatment for breast cancer. Supportive Care in Cancer. 2015 Jul;23(7):2061-2071.

28. Wang YJ, Boehmke M, Wu YW, et al. Effects of a 6-week walking program on Taiwanese women newly diagnosed with early-stage breast cancer. Cancer Nurs. 2011 Mar-Apr;34(2):E1-13.

29. Chen Z, Meng Z, Milbury K, et al. Qigong improves quality of life in women undergoing radiotherapy for breast cancer: results of a randomized controlled trial. Cancer. 2013;119(9):1690-1698.

30. Zhou K, Wang W, An J, et al. Effects of progressive upper limb exercises and muscle relaxation training on upper limb function and health-related quality of life following surgery in women with breast cancer: a clinical randomized controlled trial. Annals of Surgical Oncology. 2019;26(7):2156-2165.

31. World Bank – processed by Our World in Data. Average years of schooling for women [dataset]. World Bank, World Bank Education Statistics (EdStats) 2023 [original data] 2023 [cited 2023 7 November]. Available from: <https://ourworldindata.org/grapher/mean-years-of-schooling-female?tab=table&time=2004..2005>

32. World Bank – processed by Our World in Data. Average years of schooling for women [dataset]. World Bank, World Bank Education Statistics (EdStats) 2023 [original data]. 2023 [cited 2023 7 November]. Available from: <https://ourworldindata.org/grapher/mean-years-of-schooling-female?tab=table&time=2006..2009>

33. OECD. Educational attainment and labour-force status: Trends in educational attainment, by educational attainment and age group: OECD.Stat; 2023 [updated 07 Nov 2023; cited 2023 7 November ]. Available from: <https://stats.oecd.org/Index.aspx?QueryId=93191>

34. Ministry of Education, Republic of China (Taiwan). Enrollment Rates of School - Total Net Enrollment Rates: Ministry of Education 2022 [updated 22-05-04; cited 2023 7 November]. Available from: <https://stats.moe.gov.tw/files/ebook/indicators/13.pdf>

35. Kim C-J, Kang D-H, Smith BA, et al. Cardiopulmonary responses and adherence to exercise in women newly diagnosed with breast cancer undergoing adjuvant therapy. Cancer nursing. 2006;29(2):156-165.

36. DeNysschen CA, Brown JK, Cho MH, et al. Nutritional Symptom and Body Composition Outcomes of Aerobic Exercise in Women With Breast Cancer. Clinical Nursing Research. 2011 Feb;20(1):29-46.

37. United Nations. Department of Economic and Social Affairs Population Division: Estimates and Projections of Women of Reproductive Age Who Are Married or in a Union 2022 [cited 2023 7 November]. Available from: <https://population.un.org/dataportal/data/indicators/44/locations/840,124,156,158,250,528,826,724,208,752,76/start/2000/end/2020/table/pivotbyvariant>

38. Swenson KK, Nissen MJ, Anderson E, et al. Effects of exercise vs bisphosphonates on bone mineral density in breast cancer patients receiving chemotherapy. J Support Oncol. 2009 May-Jun;7(3):101-7.

39. Stan DL, Kathleen Sundt R, Cheville AL, et al. Pilates for breast cancer survivors: Impact on physical parameters and quality of life after mastectomy. Clinical journal of oncology nursing. 2012;16(2):131.

40. Dieli-Conwright CM, Parmentier J-H, Sami N, et al. Adipose tissue inflammation in breast cancer survivors: effects of a 16-week combined aerobic and resistance exercise training intervention. Breast Cancer Res Tr. 2018;168(1):147-157.

41. Lee K, Kang I, Mack WJ, et al. Feasibility of high intensity interval training in patients with breast Cancer undergoing anthracycline chemotherapy: a randomized pilot trial. BMC cancer. 2019;19(1):1-9.

42. Statistics Canada. Canada (Code 01) (table). National Household Survey (NHS) Profile. 2011 National Household Survey. Statistics Canada Catalogue no. 99-004-XWE. Ottawa. Released September 11, 2013. Statistics Canada2013 [cited 2023 29 November]. Available from: <https://www12.statcan.gc.ca/nhs-enm/2011/dp-pd/prof/details/page.cfm?Lang=E&Geo1=PR&Code1=01&Data=Count&SearchText=Canada&SearchType=Begins&SearchPR=01&A1=All&B1=All&Custom=&TABID=1>

43. Statistics Canada. Canada [Country] and Canada [Country] (table). Census Profile. 2016 Census. Statistics Canada Catalogue no. 98-316-X2016001. Ottawa. Released November 29, 2017. 2017 [cited 2023 29 November]. Available from: <https://www12.statcan.gc.ca/census-recensement/2016/dp-pd/prof/details/page.cfm?Lang=E&Geo1=PR&Code1=01&Geo2=PR&Code2=01&Data=Count&SearchText=canada&SearchType=Begins&SearchPR=01&B1=All&TABID=1>

44. Statistics Canada. Visible minority and population group by generation status: Canada, provinces and territories, census metropolitan areas and census agglomerations with parts. Table: 98-10-0324-01. Released 2022-10-26. 2023 [cited 2023 29 November]. Available from: <https://www150.statcan.gc.ca/t1/tbl1/en/tv.action?pid=9810032401>

45. Statistics Canada. The Canadian census: A rich portrait of the country's religious and ethnocultural diversity Statistics Canada2022 [cited 2023 29 November]. Available from: <https://www150.statcan.gc.ca/n1/daily-quotidien/221026/dq221026b-eng.htm>

46. United States Census Bureau. "Profile of General Demographic Characteristics: 2000." Decennial Census, DEC State Legislative District Demographic Profile (100-Percent), Table DP1, 2000 2000 [cited 2023 29 November]. Available from: <https://data.census.gov/table?y=2000>

47. United States Census Bureau. Race and Ethnicity in the United States: 2010 Census and 2020 Census 2021 [cited 2023 29 November]. Available from: <https://www.census.gov/library/visualizations/interactive/race-and-ethnicity-in-the-united-state-2010-and-2020-census.html>
